# A Larger Chocolate Chip—Development of a 15K *Theobroma cacao* L. SNP Array to Create High-Density Linkage Maps

**DOI:** 10.3389/fpls.2017.02008

**Published:** 2017-12-05

**Authors:** Donald Livingstone, Conrad Stack, Guiliana M. Mustiga, Dayana C. Rodezno, Carmen Suarez, Freddy Amores, Frank A. Feltus, Keithanne Mockaitis, Omar E. Cornejo, Juan C. Motamayor

**Affiliations:** ^1^Mars, Inc., Miami, FL, United States; ^2^Estación Experimental Tropical Pichilingue, National Institute of Agricultural Research, Quevedo, Ecuador; ^3^Agricultural Faculty, Technical University of Quevedo, Quevedo, Ecuador; ^4^Universidad Técnica Estatal Quevedo (UTEQ), Quevedo, Ecuador; ^5^Department of Genetics and Biochemistry, Clemson University, Clemson, SC, United States; ^6^Department of Biology and Pervasive Technology Institute, Indiana University Bloomington, Bloomington, IN, United States; ^7^School of Biological Sciences, Washington State University, Pullman, WA, United States

**Keywords:** cacao, SNP, genotyping, QTL

## Abstract

Cacao (*Theobroma cacao* L.) is an important cash crop in tropical regions around the world and has a rich agronomic history in South America. As a key component in the cosmetic and confectionary industries, millions of people worldwide use products made from cacao, ranging from shampoo to chocolate. An Illumina Infinity II array was created using 13,530 SNPs identified within a small diversity panel of cacao. Of these SNPs, 12,643 derive from variation within annotated cacao genes. The genotypes of 3,072 trees were obtained, including two mapping populations from Ecuador. High-density linkage maps for these two populations were generated and compared to the cacao genome assembly. Phenotypic data from these populations were combined with the linkage maps to identify the QTLs for yield and disease resistance.

## Introduction

With a preferred growing range between 10°N and 10°S of the equator, *Theobroma cacao* L. (cacao) is a diploid understory tree crop with 10 chromosomes (2n = 2x = 20). Cacao has a long history of cultural significance for both the Aztec and Mayan civilizations, with some of the earliest archeological data suggesting that cacao was used as early as 1000 BC (Henderson et al., 2007). While primarily an outbreeding crop, self-fertilization has been known to occur in certain genetic backgrounds of cacao particularly Criollo and Amelonado. The mechanism controlling self-incompatibility is only beginning to be understood (Royaert et al., 2011; Lanaud et al., 2017), however initial domestication from wild ancestors likely occurred over centuries of cultivation in Central America and Mexico contributing to the few self-compatible trees observed now (Bartley, 2005). Today, cacao remains an important cash crop in the tropics of Central and South America, Asia, and Africa (Cuatrecasas, 1964; Hunter, 1990; Motamayor et al., 2002). Prized for its seeds, commonly referred to as cocoa beans, *T. cacao* L. is the signature ingredient of chocolate and is produced in over 50 countries (Knight, 2000). The unique fat composition of cocoa beans, which are also used extensively in the cosmetics industry in the form of cocoa butter, provides a smooth texture to chocolate, while the cocoa solids provide its distinctive chocolate flavor. Due to increases in the demand for cocoa products, which have been exacerbated by increased disease pressure in its growing regions and the fact that it is mainly grown on smallholder farms, the importance of cacao germplasm improvement is more essential now than ever (Lattre-Gasquet et al., 1998; ICCO Production Values, 2012). Efforts to make cacao production more sustainable are of great importance, and building on the assembled cacao genomes to assist traditional breeding methods by developing molecular tools holds the promise of accelerating germplasm improvement toward this goal.

Colonization of the Americas led to some of the earliest attempts to cultivate cacao by the English, Dutch, and Spanish in the seventeenth century. More modern cacao breeding efforts occurred in the early 1900s (Fredholm, 1911), when cacao was classified into three major morphological/geographic groups that were still recognized up until a decade ago, when the use of genetic markers proposed a new classification of the cacao germplasm into at least 10 major groups: Marañon, Curaray, Criollo, Iquitos, Nanay, Contamana, Amelonado, Purús, Nacional, and Guiana (Motamayor et al., 2008). These groups differ greatly in both their agronomic and commercial traits, including flavor, yield, and susceptibility to diseases. The heterosis that occurs between crosses of trees from different genetic groups is exploited by the breeders to improve cacao production (Warren, 1992).

Ecuador is the leading cacao producer in the Americas, with exported cacao beans its third largest commodity (Gill, 2014). Breeding programs in Ecuador have succeeded in furthering the generation of improved cacao varieties. The foundation of our current knowledge of frosty pod and witches' broom, two devastating cacao diseases, was built upon work done in Ecuador, where *Moniliophthora roreri* was first identified as the causal agent for frosty pod and where the first reports of witches' broom disease (*Moniliophthora perniciosa*) originated (Evans et al., 1977; Gutiérrez et al., 2016). The Tropical Experimental Station in Pichilingue was established, in part, to breed for resistance to these and other cacao diseases (Evans et al., 1977). The most widely adopted cacao variety in South America, CCN-51, was developed in Ecuador by Homero Castro in the 1960s and is known for its high yield and disease resistance (Boza et al., 2013). While improved yield and resistance remain persistent breeding targets, Ecuadorian chocolate is prized by the makers of fine chocolates for its “Arriba” flavor profile. Recent efforts have focused on incorporating other desired traits, along with improved flavor profiles, while incorporating the yield and resistance alleles.

Representing distinct alleles within the genome, molecular markers represent a particular genetic location that can be associated with phenotypic traits. An important aspect of an integrated cacao genetics program is to develop and apply molecular markers for use in breeding improved varieties and curating the germplasm resources of cacao (Risterucci et al., 2000; Kuhn et al., 2003, 2008; Borrone et al., 2004; Schnell et al., 2007; Lima et al., 2009; Irish et al., 2010; Motilal et al., 2010, 2016). As sequencing technology continues to improve, identification of Single Nucleotide Polymorphisms (SNPs), which can serve as molecular markers, has substantially increased the amount of resources available to develop new cacao varieties. *T. cacao* is fortunate to have two genetically diverse genome sequence references, the Belizean Criollo genotype B97-61/B2 (Argout et al., 2011) and an Amelonado genotype, Matina 1-6 (Motamayor et al., 2013). These sequenced genomes provide a solid reference platform for SNP discovery. In cacao, SNPs have been developed and used for comparative genomic studies, genetic maps, marker assisted breeding, and determining off-types in clonal collections (Kuhn et al., 2010, 2012; Livingstone et al., 2011; Allegre et al., 2012; Cosme et al., 2016; Motilal et al., 2016).

Crop improvement efforts receive the greatest benefit when genetic markers are associated with specific phenotypic traits. The first linkage map in cacao, representing all 10 chromosomes, was produced by Lanaud et al. (1995) and it has served as the reference for naming the linkage groups of cacao. Since then, additional cacao linkage maps, utilizing a variety of marker types, have been created and used for QTL analysis to identify marker-trait associations (Pugh et al., 2004; Brown et al., 2005, 2007; Cervantes-Martinez et al., 2006; Lima et al., 2009; Royaert et al., 2011, 2016). Resolution of these QTLs is partially limited by the number of available genetic markers, as the maps were composed mostly of microsatellite markers, which have low technical precision when assayed across different equipment. SNP markers can be assayed at larger scales with lower cost compared to most other available markers. SNPs are also easily ported to different platforms, making it straightforward to compare SNP genotypes over time. In other crops, such as soybean, cotton, potato, and rice, SNP arrays have been developed and successfully used to generate linkage maps, identify QTL, track introgression, and verify pedigree (Akond et al., 2013; Cai et al., 2017; Schumann et al., 2017; Thomson et al., 2017). SNP-based linkage maps are already serving the cacao breeding community. The marker-trait associations identified in several recent cacao studies are now being used to help guide the planning of new crosses and the selection of improved varieties (Allegre et al., 2012; Motamayor et al., 2013; Livingstone et al., 2015; Motilal et al., 2016; Royaert et al., 2016).

Here we describe a new 15,000 bead-type SNP array (Illumina Infinium) that combines the sequencing variants identified from two previous projects (Motamayor et al., 2013; Livingstone et al., 2015). Selection of markers was prioritized to target gene models within the QTL regions. (Brown et al., 2005; Lanaud et al., 2009; Feltus et al., 2011; Royaert et al., 2011) To demonstrate the usefulness of the genotypes generated from this array, linkage maps and QTL analysis were performed on two large F1 mapping populations from Ecuador. Due to the dense genotype data provided by this SNP array and the relatively large population size of one of the mapping populations, our analyses identified new QTLs for agronomically important traits, and further allowed us to insert orphan sequence contigs into the cacao genome assembly.

## Materials and methods

### Diversity panel description for SNP identification

A diversity panel of 11 cacao accessions was used for SNP discovery (Table 1). The accessions were chosen because they are key clones in the breeding programs (Matina 1-6, Criollo 13, MVP30, MVT85) or parents of previously studied mapping populations (Pound 7,UF273 Type 1, UF273 Type 2, KA2-101, K82, TSH1188, CCN51). Plant material for the diversity panel was obtained from the USDA-ARS SHRS germplasm collection in Miami; the Centro Agronomico Tropical de Investigacion y Ensenanza (CATIE) in Turrialba, Costa Rica; the Cacao and Coconut Institute in Papua New Guinea (PNG); the Mars Center for Cacao Science (MCCS) in Brazil; or the Cocoa Research Institute of Ghana (CRIG) as described in Table 1. The majority of the accessions on the diversity panel come from mixed genetic backgrounds with the two predominant genetic groups presented (Table 1). In all, half of the total diversity in cacao is represented within the predominant background of these accessions.

**Table 1:** Summary of diversity panel accessions used for SNP discovery.

**Accession**	**Accession location**	**Percent heterozygosity**	**SNP sequencing source**	**Largest genetic group(s)**	**Description**
Matina 1-6	SHRS	0.1	Transcript & Genome	Amelonado	Domesticated clone
Criollo 13	SHRS	0.2	Transcript & Genome	Criollo	Domesticated clone
Pound 7	SHRS	19.8	Transcript & Genome	Nanay, Iquitos	Wild collection
UF273 Type 1	CATIE	30.5	Transcript & Genome	Nacional, Amelonado	Clonal selection
UF273 Type 2	CATIE	28.9	Genome	Nacional, Amelonado	Clonal selection
KA2-101	PNG	40.7	Genome	Amelonado, Criollo	Clonal selection
K82	PNG	41.8	Genome	Amelonado, Criollo	Clonal Selection
TSH1188	MCCS	34.3	Genome	Iquitos, Nanay	Clonal Selection
CCN51	MCCS	43.1	Genome	Amelonado, Iquitos	Clonal selection
MVP30	CRIG	1.1	Genome	Amelonado	Clonal Selection
MVT85	CRIG	24.8	Genome	Iquitos, Nanay	Clonal selection

### Transcript-based SNP identification (dataset 1)

**Figure 1:**
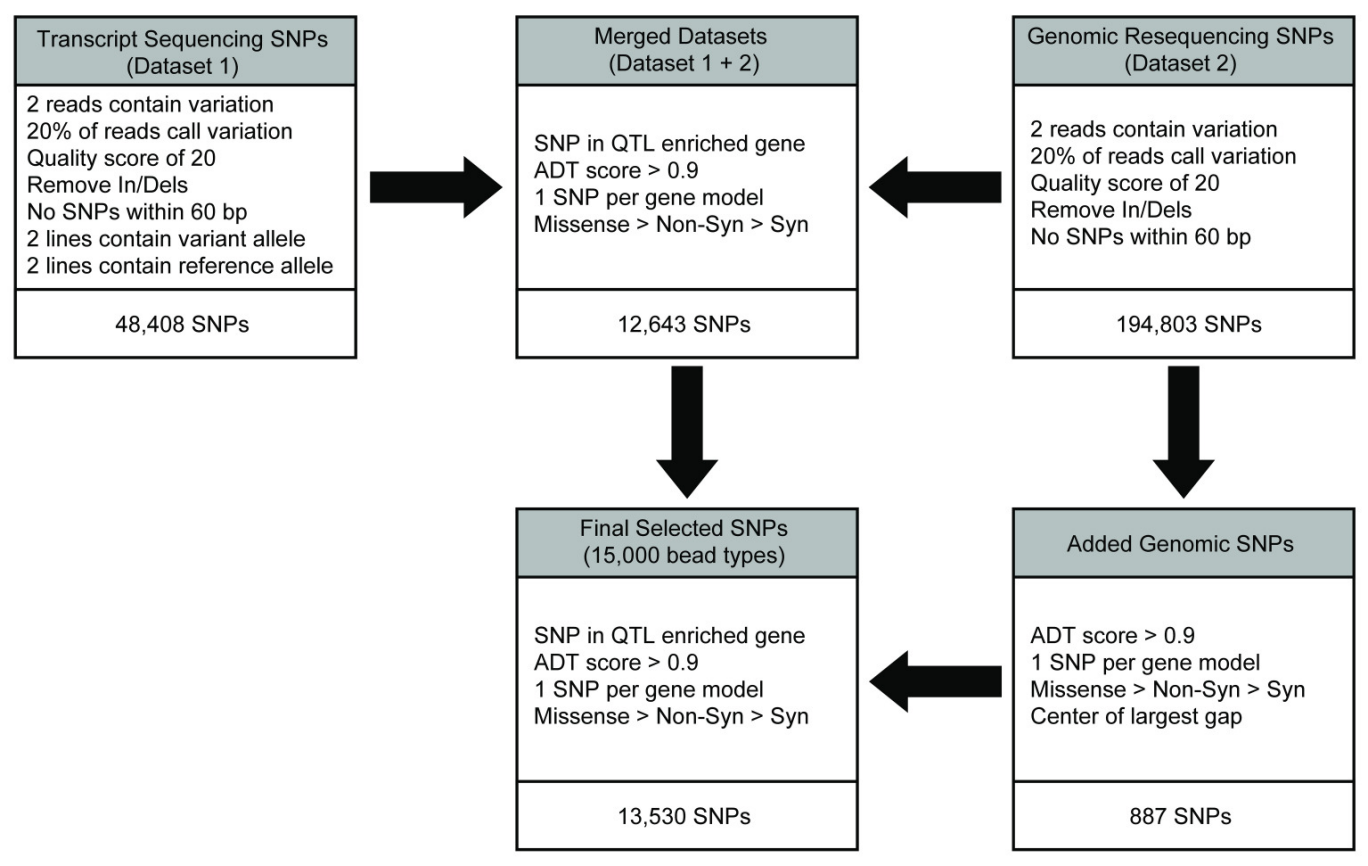
SNP discovery and filtering to select the 13,530 SNPs for inclusion on the Cacao15kSNP array. The transcript (dataset 1) and genomic resequencing (dataset 2) variants were identified by alignment to the early Matina reference genome. Initial filtering was applied to provide confidence in the existence of variants. The two variant sets were combined into the merged dataset, and filtering was applied to reduce the number of SNPs by enriching for markers within the QTL regions, identifying those SNPs most likely to perform well on the chip (ADT score), and selecting for SNPs that represented the most genes possible. SNPs were added from the genomic resequencing data (dataset 2) to reach the targeted number of markers in the final filtered SNPs for inclusion on the Cacao15kSNP array.

Transcript-based SNP calls had previously been generated for four of the diversity panel members (Table 1) as described in Livingstone et al. (2015) and it contains 48,408 filtered SNPs. In short, leaf RNA samples isolated from each accession were sequenced on the Illumina GAII platform and aligned to the *T. cacao* 454 sequencing derived from the Matina 1-6 leaf transcriptome (Livingstone et al., 2015). The SNPs were identified and filtered as follows: variant bases had a Phred-type quality score of Q20 or higher, an observed frequency of 20% or higher at the position calling the variant allele, and two or more sequence reads calling the variant. Non-bivariate SNPs and In/Dels were also removed. A total of 48,408 filtered SNPs were identified in this manner, designated as dataset 1, used for SNP selection on the Cacao15kSNP array (Figure 1).

### Genome-based SNP identification (dataset 2)

Genomic DNA from each of the 11 diversity panel members was resequenced, aligned to the Matina 1-6 reference genome, and the sequence variants were identified (Motamayor et al., 2013). The same initial filtering for dataset 1 was also applied here: variant bases had a Phred-type quality score of Q20 or higher, a frequency of 20% or higher at the position calling the variant allele, and two or more sequence reads calling the variant. In/Dels were removed, as were any SNPs within 60 bp of another variant to ensure proper genotyping on the Cacao15kSNP array (Figure 1). The 194,803 filtered SNPs identified in this manner are designated as dataset 2.

### QTL-enriched gene models

The gene model annotation from the Matina 1-6 genome (Motamayor et al., 2013) was used to create a list of enriched gene models that are present within the QTL regions. The genomic position of the published QTL regions based on microsatellites (Borrone et al., 2004; Brown et al., 2005, 2007; Lanaud et al., 2009; Feltus et al., 2011; Royaert et al., 2011) was used in conjunction with the Matina 1-6 gene models to identify all the genes that fall within those QTL regions. By looking for the enrichment of discrete functions [e.g., Gene Ontology (GO), Protein Domains (INTERPRO), Enzyme (EC) & Pathway (KEGG) annotations] in the summed QTL space for a trait relative to the genome background of functional term counts, it was possible to scan for significant, QTL-enriched functional annotations and the genes that code for that function. The expectation is that each of these candidate genes would have a higher probability of driving the QTL effect and should therefore be higher priority targets for polymorphism discovery.

### Final SNP selection

**Figure 2:**
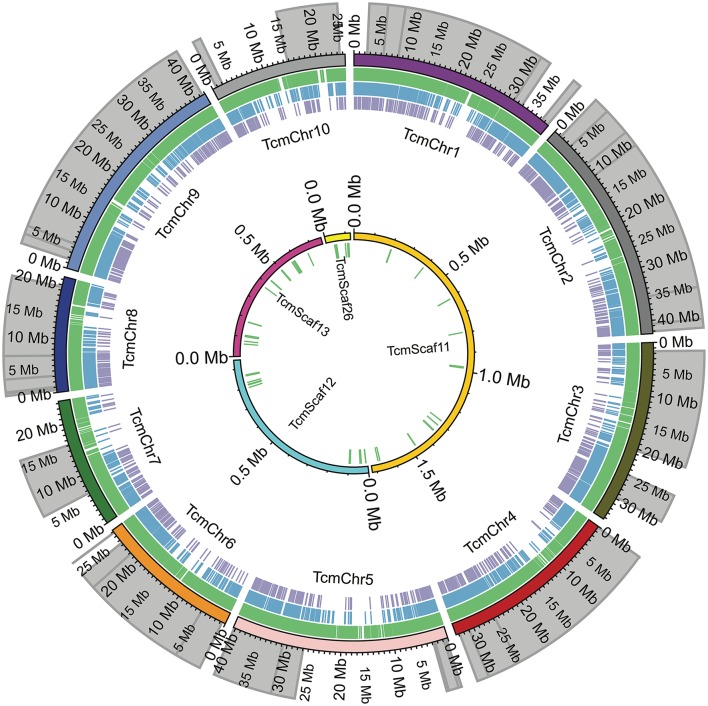
Comparison of SNP locations in the Matina 1-6 genome. The chromosomes (TcmChr) and unanchored scaffolds (TcmScaf) of the Matina 1-6 v1.1 genome assembly are presented as concentric circles composed of colored arcs with their physical size in Mbp indicated. The outer circle comprises the 10 chromosomes, while the unanchored scaffolds are the inner circle. The location of the enriched QTL regions used in SNP selection are shaded in gray. Colored tick marks represent the SNPs selected for inclusion on the Cacao15kSNP array (green tick marks), SNPs present on the Cacao6kSNP array (blue tick marks), and SNPs in common between the two arrays (purple tick marks).

Filtered SNP datasets 1 and 2 were merged, keeping only the SNPs common to both, and then an additional round of filtering was performed on the combined dataset. One thousand SNPs from the combined dataset that were segregating in at least one of the three mapping populations genotyped with the Cacao6kSNP array (Livingstone et al., 2015) were chosen to serve as anchor markers to link the data from the previous Cacao6kSNP array and Cacao15kSNP array described here. Preference was given to the markers in an enriched gene model or the markers that were observed to be polymorphic within all three previously genotyped mapping populations. To make the number of anchor markers from each chromosome proportional to the number of predicted gene models in that chromosome, markers were then removed at random (where they were overrepresented) or were added at random from the combined data set (where they were underrepresented; Figure 2). SNP selection continued by reducing the merged dataset (Figure 1). First, the SNP sequences were sent to Illumina for Assay Design Tool (ADT) scoring, which is a measure of the likelihood of success for a potential probe sequence based on the GC content and sequence adjacent to the SNP site. A value from zero to one, with 1 being the most likely to succeed, is assigned and only those SNPs with an ADT score greater than 0.9 were kept. Next, the SNPs within the QTL-enriched gene model list were selected. Previously, two SNPs per transcriptome contig had been the target to ensure transcript representation, but this requirement proved to be redundant as the marker success was high (Livingstone et al., 2015). For the Cacao15kSNP array, we targeted one SNP per QTL-enriched gene model. If more than one SNP existed with a gene model, we favored, in decreasing order, those SNPs with unique flanking regions (non-duplicated flanking regions), SNPs that were predicted to produce a missense or non-synonymous amino acid change (according to alignments to the reference genome; Motamayor et al., 2013), SNPs using a single bead-type (A/C, A/G, T/C, T/G), and lastly, SNPs with high ADT scores. This filtering approach resulted in slightly less than the required 15,000 bead-types (Figure 1). To increase the number of bead-types to 15,000, additional SNPs from dataset 2 that met all of the above listed filters were selected by filling in the largest gaps present between any two selected SNPs. The final list of 15,000 bead-type variants, representing 13,530 SNP sequences (12,643 gene models), was submitted to Illumina for Infinium SNP Array production and subsequent genotyping (Supplementary Table 1). A summary of the SNPs selected for inclusion on the array is presented in Table 2.

**Table 2:** Summary of the SNP content present on the array.

**Scaffold**	**Number of SNPs**	**SNPs within gene models**	**Average distance between SNPs (bp)**	**Max gap between SNPs (bp)**	**Min gap between SNPs (bp)**
1	1,595	1,496	24,441	796,601	109
2	1,727	1,620	24,583	582,845	637
3	1,386	1,323	24,801	466,526	113
4	1,382	1,301	24,248	507,221	74
5	1,651	1,547	24,490	669,008	539
6	1,115	1,041	24,401	522,006	371
7	977	903	24,945	722,324	454
8	873	831	24,622	573,082	473
9	1,729	1,612	24,286	856,591	271
10	999	929	25,450	908,235	284
11	30	13	51,345	302,703	21
12	24	7	37,234	699,415	81
13	32	15	19,641	202,236	77
26	10	5	8,378	45,897	81
all	13,530	12,643	25,919	908,235	21
LG 1-10	13,434	12,603	24,627	908,235	74

### Plant material for genotyping and linkage mapping

Leaf samples were collected from a total of 3,072 cacao trees at INIAP, Ecuador. The DNA from these samples was extracted using the Zymo Research plant DNA extraction kit, following the manufacturer's protocol (Zymo Research, Irvine, CA), and were submitted to Illumina for genotyping on the custom Infinium II BeadArray. The trees that were sampled came from the following populations / breeding trials: EET 95 × Silecia 1 (Stalin A) mapping population (733 individuals, including both parents), Scavina 12 × Unknown (Stalin B) mapping population (251 individuals, including the single, known parent), representatives of the clones from the Tecas (655 individuals), Ganaderia (409 individuals), and Malvinas field (401 individuals) clonal breeding trials, along with a sampling of their progenitors (66) from INIAP, Ecuador. Additional samples were collected from the Trinidad germplasm collection (136 individuals), the L12 progeny trial, with parents at CATIE (151 individuals), and selected clones from the SHRS, MCCS, and MRCC germplasm collections (172 individuals). The remaining 98 samples were internal control samples from the Matina 1-6, Criollo 13, or Pound 7 trees located in Miami.

### SNP array genotype processing

Data generated from all 3,072 samples were analyzed with GenomeStudio (V2011.1, Illumina) using the Genotyping module (1.9.4, Illumina) and SNP clustering was performed, as part of the Illumina Genotyping module, with the custom GenTrain clustering algorithm that incorporates several biological heuristics. The genotype data were filtered to remove the SNP markers that failed to generate data for any sample. Additionally, data points with low GenTrain score (<0.4) and GenCall score (<0.2) scores were removed. GenTrain score is a statistical score that evaluates clustering based on the shape of the clusters, their relative distance to each other, and mimics evaluations made by human experts. GenCall score is a quality metric ranging from 0 to 1. The lower the value the farther away from the center of the cluster a data point is; values below 0.15 are considered no calls. The data were exported as a whole, and subsets of the Stalin A and Stalin B mapping populations were generated.

### F1 mapping populations

The Stalin A mapping population consisted of 731 F1 full-sib progeny from a cross between two parents, EET 95 and Silecia 1. The mother, EET-95, is a heterozygous (90.1%), high-yielding clone that is an F1 from an open-pollinated Nacional-type mother. It is often known for providing the fine flavor characteristics of Ecuadorian chocolate. The father, Silecia 1, is a homozygous (79.5%) cultivar from the upper region of the Amazon and has been shown to be tolerant to witches' broom (Eskes, 2010). This cross was made in 1968 as part of a breeding effort to improve yield and disease resistance (Hardy, 1960; Hunter, 1961; Rudgard et al., 1993; Eskes, 2010). The Stalin B mapping population comprises 250 F1 full-sib progeny from a cross between two parents, Scavina 12 (mother, 50% heterozygous) and a single, but unknown, father (58.8% heterozygous). The father tree was either not characterized or this information has been lost. The tree had reportedly died before the start of this study, but it was likely a high-yielding selection from a local farm. Scavina 12 is known to carry resistance to witches' broom (Eskes, 2010). From Stalin A, both parents and 705 out of the 731 progeny samples were successfully genotyped. From Stalin B, the mother and 249 out of the 250 progeny samples were successfully genotyped. Two additional progeny were removed from Stalin B because their genotypes were identical, and they clearly represented a mistake during leaf collection or sample processing. A genotype for the unknown father of Stalin B was inferred from allelic segregation in the progeny and the genotype of the mother. This was done for each locus by calculating the chi-squared goodness of fit of the genotype segregation against the hypothetical genotype segregation patterns for a population of F1 progeny, assuming Mendelian inheritance and given the maternal genotype. The model with the smallest chi-squared test statistic was assumed to represent the true segregation pattern at a locus, and the father genotype was assigned accordingly. The scripts for this inference can be found in the Supplemental Material (Supplementary File 1).

Plink version 1.9 (Chang et al., 2015) software was used to calculate the frequency of Mendelian errors in the progeny genotypes of both Stalin A and B, which, in the latter case, used the inferred father (Supplementary File 1). Progeny where 3% or more of the genotyped loci violated Mendelian inheritance were considered to be off-types. A total of 129 progenies from the Stalin A cross and 9 from the Stalin B cross were identified as off-types and were excluded from further analysis.

### Development of linkage maps

Genetic linkage maps were created for the two F1 populations using JoinMap (version 4.1; Kyazma BV, Wageningen, the Netherlands; Stam, 1993; Van Ooijen, 2011). All members of the mapping populations described above were genotyped for the 13,530 SNPs, but not all of the SNPs segregated in the mapping populations. The resulting SNP genotype data were manually transformed to include only those markers segregating in the progeny and were formatted for use with the JoinMap software (Stam, 1993). The maps for both Stalin A and B were created in JoinMap using the outbreeding, full-sib family (“CP”) population type. The following rules were maintained to provide robust genotype data: any SNP loci that had missing values for more than 5% of the trees, along with SNP markers showing high Chi (Royaert et al., 2011) values (>15), were removed from the analysis, and finally, individual trees that were missing data for more than 15% of the SNP markers were removed. Additionally, the similarity of the loci was examined and identical loci were excluded, keeping only the first listed marker. Identical markers will map to the identical map position, and removing them results in faster calculations (JoinMap Manual). Likewise, trees with identical genotypes across every marker were removed from all analyses (two individuals from Stalin B). The maps were made using the maximum likelihood (ML) mapping algorithm with the following changes to the default settings: set recombination frequency start to 0.5 and set recombination frequency end to 0.1. After an initial mapping was performed for each linkage group, the Fit and Stress values for the markers were examined. Any marker with a Fit and Stress value, a measure of uncertainty in placement, above 2 was removed, and the map was remade until no marker showed high stress values (Kyazma/Johan W. Van Ooijen personal communication). For each of the linkage groups, the ordering of the maps was calculated via JoinMap's Metropolis-Hasting algorithm as part of a Gibbs sampler run with random starts. Since the ordering can depend on the proposed random start value, the maps were calculated several times until the most consistent version of the map was obtained as a way to check convergence, provided that no high fit and stress values were present. For each population, the maps were first constructed by linkage group and by parental segregation types (lmxll, nnxnp, hkxhk). For each linkage group, four separate maps were created, which consisted of markers that segregate only in the mother (lmxll), markers segregating only in the father (nnxnp), markers that segregate in both parents (hkxhk), and all the markers for a particular LG. The positions of the markers in each map was compared to the overall map and checked for collinear marker order. The final maps selected were those with best collinear agreements.

### Phenotypic data collection

The F1 progenies from both populations were planted at 4 × 4 m in 1969 in Lot 7A at INIAP. The parents of the trials are planted as clones at a different lot as part of a germplasm collection, except for the unknown father from Stalin B. The experimental site is at 75 m above sea level. Annual temperature and rainfall averages for the last 25 years are 24.9°C and 2166 mm. There is a seasonal pattern of rainfall with wet season in the first half of the year and a dry season in the second half of the year. Although data collection was discontinued for long periods, in 2002, trees were pruned to obtain uniformity, making it possible to start data collection on a monthly basis during 2003–2008, which is the dataset used in these analyses.

Phenotypic data were collected on both mapping populations by field workers at INIAP over the course of ~5.5 years (January 2003–July 2008). For each tree, the number of healthy pods, number of pods infected with Frosty Pod (FP) rot (*M. roreri*), number of sick pods (not including those with FP), number of wilted cherelles (dried, aborted fruits still attached to the tree), and wet bean weight (grams) were observed at ~30-day intervals. Counts of the number of trunk cankers (multiple etiological agents) and the incidence of witches' broom (WB) disease (*M. perniciosa*) were made annually, typically in July or August. Incidences of WB were counted for three classes of plant tissue: vegetative growth (Vegetativas), flower cushions (Coginete), and pods (Chirimoya). For the traits, broad-sense line-mean heritabilities were calculated with Genstat software (VSN International, 2015) with the method described in Cullis et al. (2006). In addition, for each trait, the genotype, year, and genotype by year effect was evaluated with two generalized linear models; one model that assumes binomial distribution for traits involving ratios (e.g., percentage of pods infected monilia) and the second model assumes Gaussian distributions otherwise.

### QTL mapping

QTL mapping analysis was performed on the final linkage maps, and the phenotype statistics were summarized using Genstat (VSN International, 2015). Monthly phenotypic measurements were summed over the data collection period, and these aggregates and means were used to create the response variables for Genstat. Each trait was mapped separately, first by using the Interval Mapping (IM) procedure, followed by Composite Interval Mapping (CIM) with cofactor selection. CIM was used with the aim to narrow down the QTL region and break apart the correlations, if any, between the markers of largest effects (peak) with surrounding markers. Hence, the markers of largest effect from the IM are used as cofactors in every iteration. To test the consistency of the QTL candidates, CIM was run several times on every trait; however, no additional changes in QTL candidates were seen after a maximum of two iterations of CIM. The threshold for significance was set to the default, corrected-Bonferroni option in Genstat, as described in Li and Ji (2005), with a 0.05 significance level. The traits of interest in both the Stalin A and Stalin B populations consisted of disease resistance and yield. More specifically, the traits included the aggregate counts per year over the course of the trial; the means of witches' broom disease that affected the flowers (cushion brooms), developing pods (chirimoya), and branches (vegetative brooms); and the counts of total pods, pods infected with frosty pod, and sick pods. Sick pods are pods that are affected by a pathogen. The percent of sick pods and the percent of frosty pods are ratios that are relative to the total pods. The cherelle wilt ratio is the number of cherelles relative to the potential yield, which is defined by the sum of the cherelle counts and total pods. The probability of the monilia infected pods was calculated as the number of observations in which at least one pod was infected with monilia, divided by the total number of times pods that were present.

## Results

### Filtering and SNP statistics

SNPs were identified across a panel of 11 genotypes representing parents of the mapping populations and select clones and were based on a combination of two separate datasets. Figure 1 shows the workflow used for final selection of the SNPs ultimately leading to the inclusion of 13,530 SNPs in the Cacao15kSNP array (Figure 1). The 10 chromosomes of the Matina 1-6 genome are represented in Figure 2 (outermost ring), with the enriched QTL regions shaded in gray. SNPs selected for inclusion on the Cacao15kSNP array are indicated (green tick marks) in relationship to those present on the Cacao6kSNP array (blue tick markers) and SNPs in common between the two arrays (purple tick marks). The inner rings (TcmScaf11-13, 26) represent some of the largest unanchored genome assembly scaffolds of the Matina 1-6 assembly (Figure 2, inner), with the SNPs included on the Cacao15kSNP array also presented as green tick marks. Assembly scaffolds 1-10 represent the 10 chromosomes of cacao respectively. The inclusion of SNPs from unanchored genome assembly scaffolds aimed to place these scaffolds onto the 10 cacao chromosomes.

### SNP array evaluation

The number of successfully synthesized markers on the array amounted to 11,930 out of the 13,530 originally submitted SNPs, which represents an 88% successful conversion rate. SNPs that failed to produce genotype data for any sample (951), along with those with low GenTrain (<0.4) and GenCall (<0.2) scores, were removed prior to analysis. Ultimately, data were obtained for 10,688 SNPs. The number of DNA samples submitted for genotyping on the Cacao15kSNP array totaled 3,072, including replicated controls. Only 34 DNA samples failed to provide any genotype information. In total, 32,470,144 data points were obtained (10,688 SNP × 3,038 DNA samples) out of a possible 36,648,960 data points (11,903 SNPs × 3,072 samples), representing 11% missing data.

### Comparison of allele calls from chip array and sequencing

An examination of the allele calls generated in the unfiltered sequencing datasets that were used to select SNPs and the genotype data generated by the Cacao15kSNP array were performed. Six of the clones used in the diversity panel were placed on the Cacao15kSNP array as controls, and the genotype calls from the array are compared to the genotype calls obtained from sequencing during SNP discovery in Table 3. The right-hand side of Table 3 shows a contingency table for each of the six varieties, comparing the count of genotype calls made by the Cacao15kSNP Array (rows) and the genotype calls made from the short-read sequencing that we used to identify the SNPs. The comparisons were broken down in to three classes based on whether the calls were “Homozygous Reference,” indicating that the alleles at a locus were homozygous for the Matina 1-6 reference allele; “Homozygous Variant,” indicating that the alleles at a locus were homozygous for the non-Matina 1-6 reference allele; or “Heterozygous,” indicating that one of the two alleles at a locus matched the allele from the reference genome, while the other allele did not match the reference allele. These six clones represent the two sequenced genomes (Matina 1-6 and Criollo 13), as well as the parents from two of the mapping populations run on the Cacao6kSNP array (TSH 1188, CCN 51, Pound 7, and UF273 Type 1). The comparison consisted of 63,543 total data points, of which 95% did not vary between the diversity panel sequencing and the array genotype calls (Table 3). This value is slightly better than the concordance found in the Cacao6kSNP array (Brown et al., 2007). The identical genotype calls within a particular clone range from 89% (TSH 1188) to 99% (Matina 1-6 and Criollo 13). The genotype calls from the Cacao15kSNP array can be observed vertically in Table 3, while the genotype determined by sequencing is shown horizontally for each clone. Concordant calls can be found along the diagonal from upper left to lower right.

**Table 3:** Comparison of the Cacao15kSNP array and sequencing calls across common accessions.

**Clone**	**Concordant SNPs**	**Non-concordant SNPs**	**Percent concordance (%)**			**Sequencing genotypes**		
						**Homozygous Reference**	**Heterozygous**	**Homozygous Variant**
				Cacao15kSNP array genotypes	Homozygous reference	10,604	9	0
Matina 1-6	10,604	39	99		Heterozygous	11	0	0
					Homozygous variant	19	0	0
					Homozygous reference	2,114	10	14
Criollo 13	10,502	73	99		Heterozygous	10	5	11
					Homozygous variant	22	6	8,383
					Homozygous reference	4,425	25	1
TSH 1188	9,328	1,137	89		Heterozygous	805	3,305	258
					Homozygous variant	35	13	1,598
					Homozygous reference	4,015	22	1
CCN 51	9,639	943	91		Heterozygous	693	4,417	198
					Homozygous variant	17	12	1,207
					Homozygous reference	6,648	11	1
Pound 7	10,438	197	98		Heterozygous	139	2,079	19
					Homozygous variant	22	5	1,711
					Homozygous reference	4,069	20	4
UF273 type I	9,998	645	94		Heterozygous	479	3,069	102
					Homozygous variant	26	14	2,860

### Linkage mapping

Subsets of the data from the Cacao15kSNP array that represent markers segregating in either the Stalin A (EET 95 × Silecia 1) or Stalin B (Scavina 12 × unknown) mapping populations were generated. Only SNP markers that are heterozygous for at least one parent within these F1 populations were retained for mapping, while uninformative (identical or opposing homozygous) markers were removed. SNP markers that segregate in at least one of the mapping populations genotyped on the Cacao6kSNP array were chosen for inclusion on the Cacao15kSNP array to serve as anchor markers (Livingstone et al., 2015). The markers that are common across maps may be used as anchor points to combine the linkage maps from different populations. Stalin A contains 6,202 segregating SNPs, while Stalin B, with its unknown parent, contains 3,636 segregating SNPs. The two F1 mapping populations share 2,603 segregating SNP markers. Additionally, 909 anchor markers segregate in the two populations (393 in Stalin A, 110 in Stalin B, and 406 in both). Although it is outside the scope of this manuscript, these 909 markers could be used to a generate a composite linkage map that combines markers from all the mapping populations used in both Cacao SNP arrays, thereby providing higher overall precision than the use of markers from any one individual map (Brown et al., 2007; Allegre et al., 2012). Included within Supplementary Table 1 is a list of which marker subset each SNP belongs to (i.e., Stalin A, Stalin B, or anchor).

**Figure 3:**
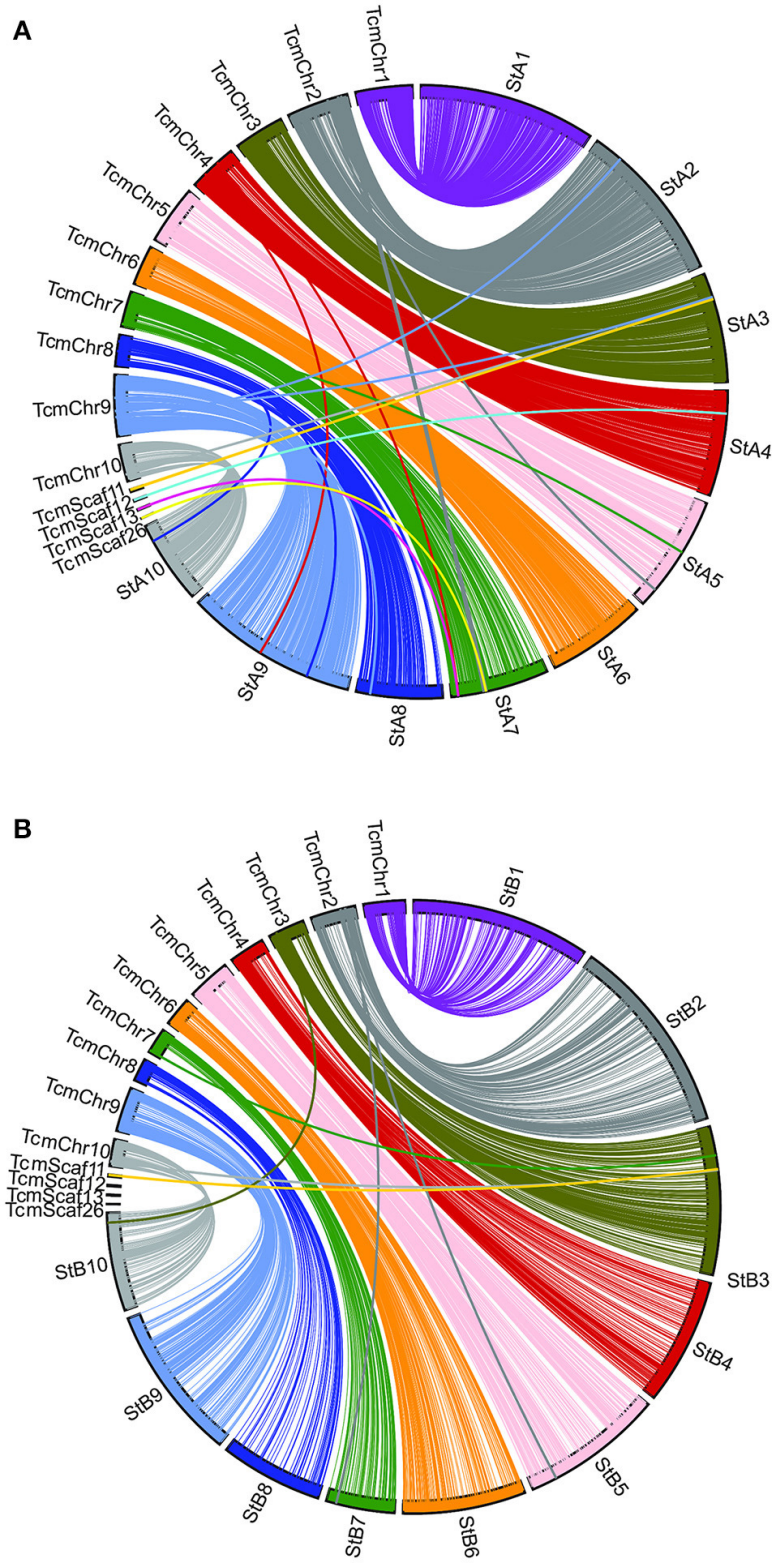
A comparison of the Matina 1-6 genome assembly and the two Stalin linkage maps. SNP marker position is compared across the Matina 1-6 genome assembly and the **(A)** Stalin A and **(B)** Stalin B genetic maps. The Stalin A linkage groups are designated by StA and the corresponding linkage group number. The Stalin B linkage groups are designated by StB and the corresponding linkage group number. Chromosomes from the Matina assembly are denoted with TcmChr. Unanchored scaffolds in the Matina assembly are identified as TcmScaf 11, TcmScaf 12, TcmScaf 13, and TcmScaf 26. Each linkage group is identified by a unique color. SNPs positioned on the Matina assembly are connected by a line to their corresponding locations within the linkage map.

Genetic linkage maps were created for both of the Stalin mapping populations using the JoinMap software (Stam, 1993; Van Ooijen, 2011), as described. Ultimately, the final linkage map of Stalin A (EET 95 × Silecia 1) was generated with 576 individuals and 3,636 SNP markers, generating a map that was 834 cM in length (Table 4). For Stalin B (Scavina 12 × unknown), a linkage map totaling 1,269 cM was created using the genotypes from 238 individuals and 1,862 SNP markers (Table 4). For both maps, the 10 identified linkage groups (LG) correspond to the 10 chromosomes of cacao, and the linkage group designations were assigned by a comparison of the mapped markers to the Matina 1-6 genome assembly, using the convention defined by Lanaud et al. (1995) and Motamayor et al. (2013). The marker order and cM position for the Stalin A and Stalin B maps can be found in Supplementary Tables 2, 3, respectively. Marker position, as determined by linkage mapping, is compared to the Matina 1–6 assembly in Figure 3.

**Table 4:** Summary of the generated Stalin A (top) and Stalin B (bottom) linkage maps.

**LG**	**No. of SNPs**	**Length (cM)**	**Largest gap length (cM)**	**Length (bp)**	**Genome coverage (%)**	**SNPs/cM**	**bp/cM**	**bp/SNP**
**STALIN A**								
LG01	495	121.8	1.6	38,988,864	99.2	4.1	313,363	78,113
LG02	544	111.6	1.6	42,436,413	99.7	4.9	374,962	77,795
LG03	431	75.5	1.2	34,397,752	99.6	5.7	468,871	79,510
LG04	413	72.5	1.2	33,492,547	99.5	5.7	450,916	80,687
LG05	242	81.1	7.3	40,442,896	98.3	3.0	491,822	164,250
LG06	285	68.5	2.1	27,290,986	99.1	4.2	391,050	94,934
LG07	229	67.5	2.9	24,379,470	99.7	3.4	340,640	106,171
LG08	205	59.4	6.9	21,543,242	96.8	3.5	346,175	101,678
LG09	512	119.1	2.3	42,035,188	99.4	4.3	338,604	81,601
LG10	280	57.4	1.8	25,448,839	98.9	4.9	411,479	89,881
Total	3,636	834.5	2.9	330,456,197	99.0	4.4	392,788	95,462
**STALIN B**								
LG01	206	168.7	7.1	38,988,864	99.1	1.2	226,224	187,595
LG02	207	188.3	10.0	42,436,413	99.2	1.1	222,195	203,299
LG03	272	139.9	3.8	34,397,752	99.3	1.9	252,969	125,544
LG04	153	119.8	6.0	33,492,547	99.1	1.3	273,066	216,831
LG05	274	134.8	6.9	40,442,896	99.2	2.0	295,987	146,402
LG06	193	116.0	5.8	27,290,986	99.3	1.7	231,031	140,455
LG07	87	64.7	5.4	24,379,470	49.6	1.3	355,440	139,089
LG08	73	97.0	7.3	21,543,242	93.5	0.8	212,007	275,877
LG09	238	146.7	10.1	42,035,188	98.5	1.6	274,956	173,973
LG10	159	93.0	5.4	25,448,839	99.0	1.7	254,200	158,422
Total	1,862	1,268.8	6.8	330,456,197	93.6	1.5	259,808	176,749

### QTL mapping

**Figure 4:**
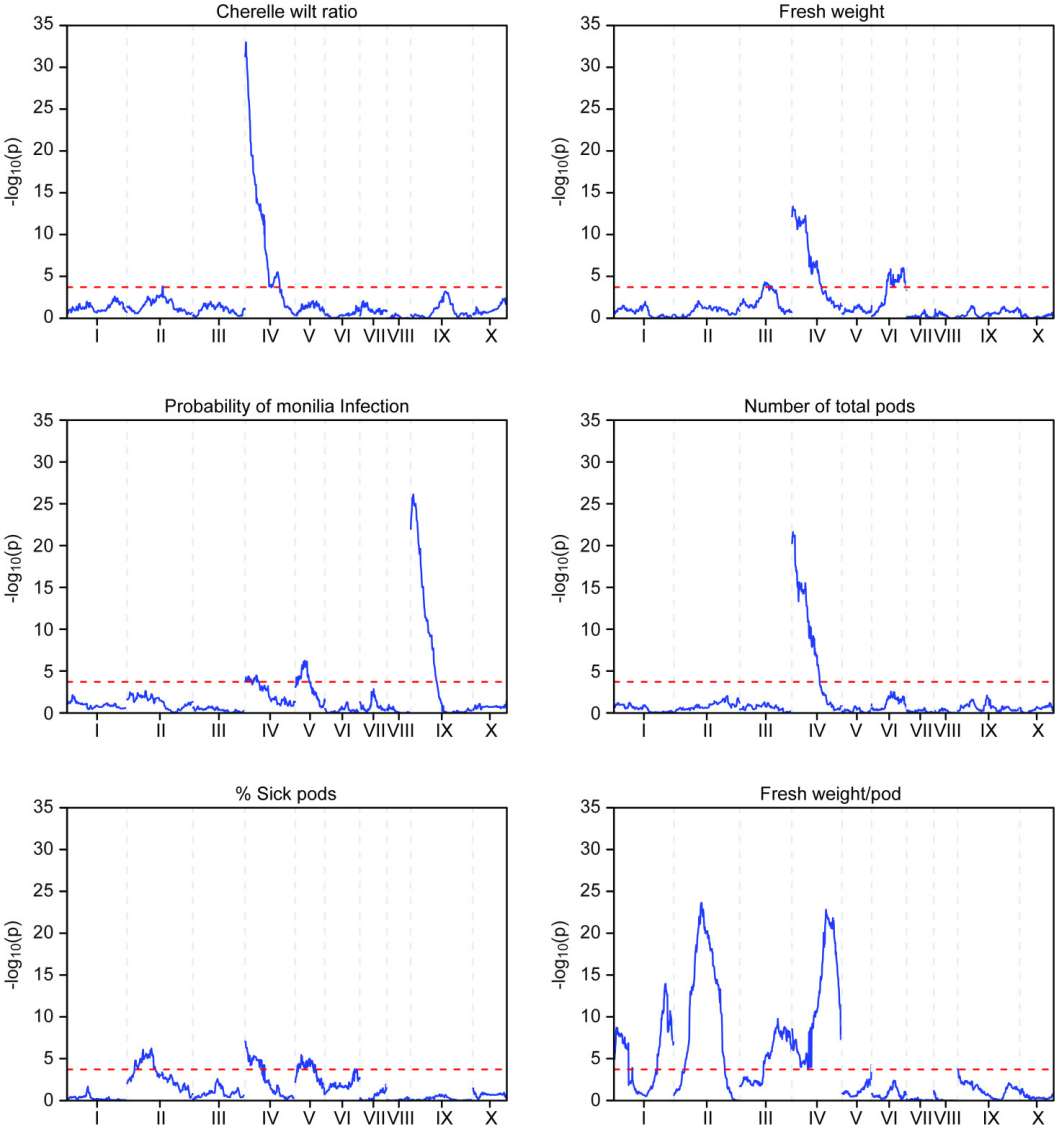
QTL mapping of the Stalin A population. Each chromosome is represented as a separate plot with the x-axis showing the marker position (cM) along the chromosome and the y-axis showing the −log_10_(p) value. Higher −log_10_(p) values indicate stronger associations. The values above a threshold −log_10_(p) value of 3.7 (dashed line) are considered significant. Six traits are represented: cherelle wilt ratio **(upper left)**, probability of monilia infection **(middle left)**, percent of sick pods **(lower left)**, fresh weight **(upper right)**, number of total pods **(middle right)**, and fresh weight per pod **(lower right)**.

**Figure 5:**
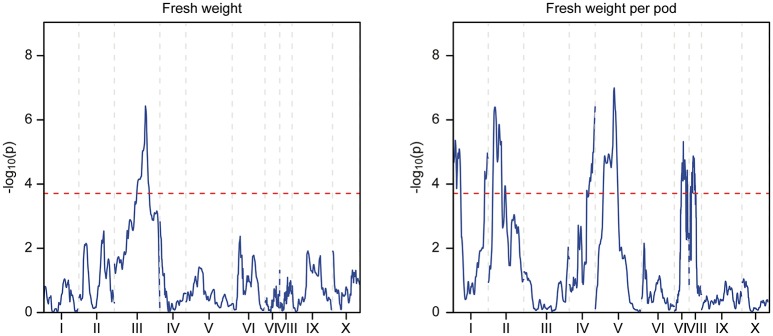
QTL mapping of the Stalin B population. Each chromosome is represented as a separate plot with the x-axis showing the marker position (cM) along the chromosome and the y-axis showing the −log_10_(p) value. Higher −log_10_(p) values indicate stronger associations. The values above a threshold −log_10_(p) value of 3.6 (dashed line) are considered significant. Two traits are represented: fresh weight **(left)** and fresh weight per pod **(right)**.

The phenotypic data collected from both the Stalin A and Stalin B populations were combined with their respective genetic maps to perform QTL mapping. The test for association is done in GenStat via mixed models with additive and dominance predictors. For each marker, the associated probability (*p*-value) from the Wald test statistic is used to test significance (Lynch and Walsh, 1998). QTLs were identified as being significant if they had a transformed *p*-value −log_10_(p) of 3.7 and 3.6 for Stalin A and Stalin B, respectively, and a percent explained greater than four. The −log_10_(p) values of the significant traits are plotted across each chromosome for Stalin A (Figure 4) and Stalin B (Figure 5). A summary of the identified QTLs is presented (Table 5), as is the allelic information for the peak markers of these identified QTLs (Table 6). Genotype by year interactions were calculated for the traits listed in Table 5. Both Stalin A and B populations had significant genotype by year interactions for percentage of monilia infected pods and fresh weight. In Stalin A, % monilia infected pods and fresh weight had significant interaction (*p* < < 0.0001, *p* = 0.0007, respectively). The Stalin B population had significant tree by year interaction for % monilia infected pods (*p* = 0.00451) and lighter effect for fresh weight (*p* = 0.0429). All other traits in Table 5 were not significant at alpha = 0.05 confidence level.

**Table 5:** Summary of the significant QTL regions in the Stalin A (EET 95 × Silecia 1) and Stalin B (Scavina 12 × Unknown) populations.

**Trait**	**Pop**	**Broad-sense Line-mean heritability**	**Method**	**Lower limit**	**Upper limit**	**LG**	**−log10 (P value)**	**% var expl**
Cherelle wilt ratio	Stalin A	0.675	CIM	366,005	469,522	4	33.0	20.0
Number of pods with Cherelle Wilt	Stalin A	0.867	CIM	350,423	469,522	4	44.96	26.29
				21,483,709	22,475,637	4	11.08	8.97
Probability of monilia infection	Stalin A	0.477	CIM	4,014,339	15,460,491	4	4.68	3.72
				2,900,378	8,370,672	5	5.97	4.24
				691,988	1,148,672	9	25.85	16.92
% of Sick pods (Non-Monilia)	Stalin A	0.431	CIM	4,520,909	4,916,911	2	6.47	5.11
				110,232	651,315	4	7.05	5.02
				2,506,854	3,708,678	5	5.44	4.78
Number of Monilia infected pods	Stalin A	0.625	CIM	300,498	1,246,640	4	12.74	9.36
				19,936,255	22,288,255	4	6.5	4.96
Proportion of Monilia infected pods	Stalin A	0.498	IM	777,617	2,162,213	9	4.86	3.72
Fresh weight	Stalin A	0.819	CIM	23,507,015	26,121,426	3	4.31	3.69
				350,423	1,286,042	4	13.36	11.10
				20,371,242	24,342,024	4	6.53	4.91
				25,226,798	26,429,152	6	6	5.08
	Stalin B	0.865	CIM	27,176,987	27,831,587	3	6.60	11.40
Number of total pods	Stalin A	0.890	CIM	300,498	469,522	4	21.64	14.92
				20,463,832	21,986,021	4	8.98	6.77
Fresh weight/pod	Stalin A	0.587	CIM	1,045,754	1,846,589	1	9.05	3.17
				35,086,841	35,627,935	1	13.97	6.1
				9,166,638	10,521,586	2	23.63	12.54
				35,480,481	35,553,943	2	8.36	4.88
				28,601,733	29,113,047	3	9.78	4.72
				110,232	469,522	4	8.54	3.18
				27,585,575	28,544,329	4	22.95	8.51
	Stalin B	0.689	CIM	2,109,609	2,661,282	1	6.45	9.18
				37,361,806	38,439,094	1	5.39	5.54
				4,915,701	5,958,246	2	6.45	7.54
				37,650,669	38,340,399	2	3.98	5.59
				32,660,090	33,359,087	4	6.32	4.43
				21,342,618	26,783,779	5	7.35	8.39
				3,067,049	4,339,051	7	5.32	5.80
				2,337,803	4,670,677	8	4.87	5.09

**Table 6:** Allele information for peak markers in significant QTL regions in the Stalin A (EET 95 × Silecia 1) and Stalin B (Scavina 12 × Unknown) populations.

**Trait**	**Pop**	**Mother**	**Father**	**LG**	**Position (bp)**	**Position (cM)**	Fav/UnFav^*^ Allele	**Mother**		**Father**	
								**H1**	**H2**	**H1**	**H2**
Cherelle Wilt Ratio	Stalin A	EET 95	Silecia 1	4	433,951	1	^*^EET95_H1 with SIL1_H1	G	A	G	A
Number of Pods with Cherelle Wilt	Stalin A	EET 95	Silecia 1	4	433,951	1	^*^EET95_H1 with SIL1_H1	G	A	G	A
				4	21,655,539	31.5	^*^EET95_H1 with SIL1_H1	G	A	A	A
Probability of Monilia Infection	Stalin A	EET 95	Silecia 1	4	8,847,274	13.6	EET95_H1 with SIL1_H1	A	G	G	G
				5	4732741	21.8	EET95_H1 with SIL1_H1	A	G	A	A
				9	837,169	4.5	EET95_H1	G	G	G	A
% of Sick Pods (Non-Monilia)	Stalin A	EET 95	Silecia 1	2	4,783,859	28.6	EET95_H1	C	A	C	C
				4	110,232	0	EET95_H1 with SIL1_H1	G	A	A	A
				5	2,566,226	12.9	EET95_H1	A	G	G	G
Number of Monilia Infected Pods	Stalin A	EET 95	Silecia 1	4	1,246,640	3.8	^*^EET95_H1 with SIL1_H1	A	G	G	G
				4	22,894,708	33.8	^*^EET95_H1 with SIL1_H1	A	G	G	G
				9	1,148,672	7	^*^EET95_H1 or SIL1_H2	A	G	G	G
Proportion of Monilia Infected Pods	Stalin A	EET 95	Silecia 1	9	602,275	2.2	EET95_H2	C	A	C	C
Fresh Weight	Stalin A	EET 95	Silecia 1	3	24,693,936	41.6	EET95_H2 with SIL1_H2	G	A	G	A
				4	433,951	1	EET95_H1 with SIL1_H1	G	A	G	A
				4	21,848,416	31.5	EET95_H1 with SIL1_H1	G	A	A	G
				6	26,041,671	57.9	EET95_H1	A	C	A	A
											
	Stalin B	Scavina 12	Unknown	3	27,438,628	91.9	SCA12_H1 or Unknown_H1	G	G	A	G
Number of Total Pods	Stalin A	EET 95	Silecia 1	4	350,423	0.7	EET95_H1 with SIL1_H1	G	A	A	A
				4	21,848,416	31.5	EET95_H1 with SIL1_H1	G	A	A	G
Fresh Weight/Pod	Stalin A	EET 95	Silecia 1	1	1,486,155	8.7	EET95_H1	A	G	G	G
				1	35,348,907	100	EET95_H2	G	A	A	A
				2	9,659,502	47	EET95_H2	T	A	T	T
				2	35,486,241	77.3	EET95_H2	G	A	A	G
				3	28,924,116	63.9	EET95_H2	C	A	A	A
				4	110,232	0	^*^EET95_H1 with SIL1_H1	G	A	A	A
				4	27,795,298	50.8	EET95_H1	G	A	A	A
	Stalin B	Scavina 12	Unknown	1	2,156,309	13.3	SCA12_H1 & Unknown_H1	A	G	A	A
				1	38,239,080	168.2	SCA12_H1	A	C	A	C
				2	5,418,786	48.7	SCA12_H1	A	A	A	C
				2	38,067,865	146	SCA12_H1, Unknown_H2	A	A	A	G
				5	24,596,934	51.7	^*^SCA12_H2	G	A	G	G
				8	3,225,622	19	SCA12_H1, Unknown_H1	C	C	A	C

* *Unfavorable allele*.

## Discussion

### Filtering and SNP statistics

Designed to focus on the previously discovered QTL regions, our filtering favored high-quality SNPs within the gene models enriched within the QTL regions (Figure 2, gray shading). For the previous Cacao6kSNP array, when possible, two SNPs were selected from each transcript in order to ensure the successful marker representation of each transcript. This led to a very high marker success rate that included redundancy. Our filtering approach for the Cacao15kSNP array reduced redundant marker representation by including only one SNP per gene model, favoring a decrease in the spacing between SNPs across the genome (Table 2). The stringent filtering of SNPs during the selection steps helped to provide true SNP markers, thereby eliminating the need for redundant markers and allowing for more coverage of the genome which will be of benefit for genome wide association studies. Of the 13,530 SNPs selected for the Cacao15kSNP array, 12,643 gene models (27.10% of all identified gene models) were each represented by a single SNP, in contrast to the Cacao6kSNP array in which 4,305 gene models (9.23% of all gene models) were represented by SNPs (Livingstone et al., 2015). In the Cacao15kSNP array the remaining SNPs derive from intergenic regions and the largest span between markers is less than 1 Mbp, found on the linkage group 10. The average distance between SNPs is ~25,000 bp, and the distribution of SNPs across each of the linkage groups is fairly even (Table 2). All the SNP markers present on the Cacao15kSNP array, their flanking sequences, position on the Matina 1-6 genome, bead-type, and possible alleles can be found in Supplementary Table 1. The number and spacing of the SNPs along the genome makes this the most saturated cacao SNP array to date. When designing an array for generalized use or to identify marker associations with previously unstudied traits, selecting markers based on even spacing along the genome may be more beneficial than selecting markers in known QTL regions.

### Comparison of allele calls from chip array and sequencing

Ninety percent of the divergent calls (2,725 out of 3,022) appear to be caused by heterozygous calls from the Cacao15kSNP array that were identified as homozygotes by sequencing. In general, the more heterozygous a clone is, the larger the amount of non-concordance is found (Table 3). For these genotype calls, the simple variant calling with the sequencing data that was used in this study can be prone to misidentifying heterozygous SNPs as homozygotes when there is low-to-median coverage (10–20x), as was the case here (Nielsen et al., 2011; Hwang et al., 2015). Although our filtering scheme tried to ensure that both alleles were called across the diversity panel, a few checks, such as examining the read coverage at a particular variant, were carried out on a single diversity panel member. This focus on exclusively the SNP metrics across the diversity panel could explain the discordant calls between the sequencing-based SNP calls of individual diversity panel members and their corresponding genotypes in the Cacao15kSNP array. If future work will rely on calling alleles based on sequencing data, or if true comparisons to other genotyping methods are to be made, the read coverage at each potential SNP in each particular sample should be controlled. In total, there were 162 instances (roughly 0.2%) of homozygote-homozygote discordance (Table 3). These discordances are likely caused by poor performance or by the misidentification of clusters within the Cacao15kSNP array.

Due in part to what is believed to be ancient domestication efforts, geographical isolation, and membership in the few genetic groups that display the ability to successfully self-pollinate, a comparison of the genotypes generated by representatives of the two sequenced cacao genomes (Criollo 13 and Matina 1-6) continue to show nearly 100% homozygosity. Matina 1-6 shows only 20 heterozygous SNPs, none of which are concordant between sequencing and the Cacao15kSNP array. Likewise, Criollo 13 has 42 heterozygous SNPs, only 5 of which are concordant (Table 3). The divergence between these clones is still apparent, with only 20% identical alleles between them, despite the nearly completely homozygous nature of both clones. We previously reported 38% shared alleles (Livingstone et al., 2015) between these two trees. The decrease in shared alleles between the Cacao6kSNP and Cacao15kSNP arrays may be the result of SNP selection. The Cacao6kSNP array targeted 2 SNPs per gene model, which are expected to be tightly linked and would lower the number of effectively independent observations for the shared allele calculation. To better understand polymorphism between these two important cacao trees, a more detailed comparative genome analysis is required. For our purpose of identifying and calling SNP variants here, however, the opposing homozygous nature of these two accessions make them well-suited as controls for the SNP genotyping reactions, as they almost always provided examples of the two differing alleles.

### Linkage mapping

The Stalin A map was built from nearly twice as many markers and individual genotypes as the Stalin B map and, as a result, is substantially smaller than the Stalin B map (Table 4). Linkage mapping relies on the identification of recombination events to place and order markers; increasing the number of individuals within a mapping population increases the likelihood of observing a recombination event between a pair of markers. The observation of less recombination events results in more co-located markers and generates gaps between groups of markers. The uncertainty associated with the order of co-located markers can lead to larger maps. These factors are probably the main contributors to the size differences between the maps based on the Stalin A and B populations. During map creation, the number of markers removed due to high Fit and Stress values was much higher for Stalin B than Stalin A, which is indicative of difficulty placing markers with confidence. Stalin A has a smaller gap size, on average, between markers (2.9 cM) than Stalin B (6.8 cM) and across all the linkage groups, with a maximum gap size of 7.3 and 10.1 cM for Stalin A and Stalin B, respectively (Table 4). The smaller gap size found in Stalin A suggests that it is the better of the two linkage maps. Furthermore, the map generated for Stalin A covers more of the Matina 1-6 assembly than the Stalin B map (99.0 vs. 93.6%, respectively). The majority of this difference in map coverage is driven by linkage group 7, which has 99.7% coverage in Stalin A, compared to only 49.6% coverage in Stalin B (Table 4). An estimation of the centimorgan size in base pairs was made, and it ranged from an average of ~259,000 bp/cM for Stalin B to 393,000 bp/cM for Stalin A. The larger estimate of bp/cM for Stalin A is consistent with the same amount of physical genome space represented within a smaller linkage map. These values are similar to those previously estimated based on the small linkage map generated from the Cacao6kSNP array (216,000 bp/cM) and the composite map created by Allegre (365,000 bp/cM) (Allegre et al., 2012; Livingstone et al., 2015). Based on the quality of the maps generated, an estimate closer to 350,000–400,000 bp/cM seems more likely. Of course, these values only represent a linear approximation for each chromosome, and the relationship between the genetic and physical positions of the markers (using Matina 1-6 as the reference genome) is more sigmoidal, as the rate of recombination increases toward the telomeres and decreases toward centromeres (Supplementary Figure 1).

A comparison of the Matina 1-6 genome assembly and the two Stalin maps was performed (Figure 3). Overall, a co-linearity is observed for a majority of the markers between their placement in the genome assembly and in the linkage maps. However, Stalin B shows some inversions and rearrangements when compared to either the genome assembly or the Stalin A linkage map (Supplementary Figure 1). These rearrangements are particularly apparent at ~50–60 cM in linkage groups 3 and 4. The observed rearrangements are likely the result of poor marker placement within the Stalin B population. There are a few instances where the markers listed on one chromosome of the genome assembly map to a different linkage group on either the Stalin A or Stalin B map. Sequence alignment of the flanking sequences of these markers to the genome assembly show a high identity to both positions within the genome assembly, perhaps suggesting duplicated sequences or regions of high homology. Such regions may have confounded the placement of these markers either in the current maps or in those that were used to originally anchor and order the genome assembly. A closer examination of the genome assembly scaffolds will be required to better understand the true placement of these markers.

During the selection of SNPs for this array, the decision was made to include markers from the genome assembly scaffolds that were not anchored onto the 10 chromosomes of the Matina 1-6 assembly (Motamayor et al., 2013). A handful of these markers were placed on one or both of the Stalin linkage maps. For example, in Stalin A, the markers from assembly scaffold 12 map to linkage group 4, and the markers from scaffolds 13 and 26 map to linkage group 7 (Figure 3). Additionally, the SNPs from scaffold 11 map to linkage group 3 in both Stalin A and Stalin B (Figure 3). By way of verification, the flanking sequence of these SNPs were blasted to a more recent Criollo assembly (Cocoa Genome Hub, 2017), which confirmed the presence of these markers in the linkage groups indicated by the linkage maps. These results demonstrate the value of a high density genetic map in ordering and improving a genome assembly.

### QTL mapping

Since both populations were created with the intent of improving the WB resistance of high-yielding breeding stock, it was surprising that no significant WB QTLs were identified in this study. The broad-sense line-mean heritability for vegetative brooms in Stalin A was 0.37 and 0.66 in Stalin B. The results for other WB traits was similar with broad-sense line-mean heritabilities for Stalin A around 0.3 and slightly higher (0.6) for Stalin B. Although the WB trait heritabilities in Stalin A are such that a QTL is not expected, the Stalin B heritabilities are higher enough that one might still expect to see QTLs. While the reasons for this are unknown, a few plausible explanations exist. In Stalin A, resistance was expected to come from Silecia 1. However, in a more recent diallele study of the General- and Specific Combining Ability of a small number of Ecuadorian clones, Silecia 1 did not have a General Combining Ability that was significantly different than zero. Unfortunately, the Specific Combining ability of EET 95 × Silecia 1 was not included in this study (Surujdeo-Maharaj et al., 2004). Additionally, our genotyping revealed replicates of many of the clones used as breeding parents at INIAP that were not genetically identical (after accounting for a 3% genotyping error), but were in fact off-types, and are improperly classified as representing a single genotype. This list included the Silecia 1 clone, which was represented by samples from two different trees whose genotypes did not match each other. Given the prevalence of off-types at INIAP, the Silecia 1 tree that was historically characterized as WB-resistant might not have been the Silecia 1 parent of the Stalin A population. In Stalin B, the known mother, Scavina 12, was recently reported to have significant resistance to WB (Surujdeo-Maharaj et al., 2004). Scavina 6 was previously identified as a source of WB resistance in Brazil (Royaert et al., 2016), and both Scavina 12 and Scavina 6 were grouped in the Contamana ancestral group (Motamayor et al., 2008). However, these two clones show different genotypes in the region identified as being associated with WB resistance in Scavina 6. This could indicate the natural diversity of WB resistance within the Contamana group. There are number of possible explanations for why no QTL was found in the Stalin B population. Stalin B is nearly half the size of the population, MP01, where the WB QTL of Scavina 6 origin was previously discovered (Royaert et al., 2016). The smaller size of Stalin B might have made our analysis under-powered, which would also explain why fewer QTL were identified in the Stalin B population relative to Stalin A. Another explanation could be that the WB strains prevalent in Brazil (where resistance was reported for Scavina 6) are pathogenically different than those in Ecuador. Indeed, Gramacho et al. (2016) found pathogenicity differences among the isolates that were prevalent in different agroecological zones within Brazil.

In the Stalin A population, significant QTLs were found for the percent of frosty pod infection, probability of frosty pod infection, total pods, number of cherelle wilt pods, cherelle wilt ratio, and fresh bean weight per pod across different linkage groups (Table 5). In Stalin A, minor QTLs for the percent of sick pods and fresh weight were found. This is not surprising, as percent of sick pods and fresh bean weight are likely caused by a number of genes that each contribute small effects to the trait as a whole. In addition, the percent of sick pods can be seen as multiple traits whose combined effect confounds the mapping.

Some of the strongest QTLs were found for potential yield, namely, total pod counts, number of pods with cherelle wilt, fresh weight, and pods infected with monilia. In terms of disease resistance, QTLs were found in Stalin A on LG09 for the percent of pods infected with monilia and the probability of monilia infection, with −log10*p*-values of 24 and 26.5 (percent variance explained of 14.8 and 17.3), respectively. The largest QTL identified in our analyses was in the Stalin A population for the number of pods with cherelle wilt, located on LG04, with a −log10*p*-value of 45 and a percent variance explained of 28.5. The number of total pods had a −log10*p*-value of 21.6 and a percent explained of 14.9. The peak markers for both of these traits were very close to each other, only 83.5 kbp apart, and the QTLs substantially overlapped with each other (Figure 4). It is plausible that these two traits are driven by (and represent) the same underlying genetic mechanism for yield, a supposition which is supported by the strong correlation (*r*^2^ = 0.72) we observed between these two traits (Supplementary Figures 2,3).

In Stalin A, the largest QTL for fresh weight per pod is on LG04 (−log10p 23.0,% var exp. 8.5%). However, in Stalin B, a significant QTL for fresh weight (−log10p 6.6, % var exp. 11.4%) was identified on LG03. Additionally, various small effect QTLs were found for fresh weight per pod in both populations (Table 5). No other significant QTLs were found for Stalin B. Both total number of pods and fresh weight per pod (0.87 correlation, Supplementary Figure 2) appear to contribute to a tree's overall yield, and it is fitting that both show strong QTLs on the same chromosome. However, while there is some overlap between the two QTLs, the highest peaks are on opposite sides of the chromosome 4 (Figure 4).

Previous QTL studies have hinted at the involvement of chromosome 4 in traits that may affect yield (Schnell et al., 2005; Yamada et al., 2010; da Silva et al., 2016); for example Royaert et al. (2011) identified a flower drop QTL on the distal end of chromosome 4 (Royaert et al., 2011). In this study, we have also observed that traits related to yield, such as the number of healthy, infected, and small pods (cherelles), are associated with this same region on LG04. These traits might all be related to the ability of a tree to generate pods, and they may not necessarily be independent of each other. This is supported by the numerous QTL for these traits that overlap on LG04 and the strong correlations seen between the traits (Supplementary Figures 2,3).

To identify the genotypic regions that are related to disease resistance and cherelle wilt, independent of the yield-related traits, we transformed these traits to remove this effect. Two of these transformations, the percent of monilia infected pods and the probability of monilia, mapped to LG09, a region that was not associated with yield in this study. However, the cherelle wilt counts and the cherelle wilt ratio (*r*^2^ = 0.11) both mapped to the same region related to yield in LG04, with similar effect sizes for each combination of parental alleles. In this case, it is not certain whether the region is capturing the incidence of cherelle wilt or if it is capturing the yield potential. It is possible that the two traits are truly controlled by two separate loci within the QTL, but the resolution of this analysis cannot distinguish them. To fully understand the cause of these traits, a much more detailed molecular analysis will be required.

## Conclusions

Here we report the development and use of a SNP-based genotyping array that represents genomic variation in *T. cacao* with high marker density. This array was built upon previous SNP panels, and it incorporated thousands of additional markers, many of which came from genes within the previously identified QTL regions. Two of the most marker-dense cacao genetic maps were created for the Stalin A and Stalin B mapping populations, and markers from the previously unanchored Matina 1-6 scaffolds were placed within the 10 chromosomes. These mappings help improve the Matina1-6 reference genome assembly. As has been documented in other studies, QTL maps can be generated using high-density, SNP-based linkage maps, and our results provide guidance for making parental or clonal selections. In addition to the example of QTL mapping demonstrated here, having more marker data can be valuable for cacao breeding efforts by allowing for more robust genomic selection and genetic diversity studies, which are currently underway with the other samples genotyped on this array. The design and analysis of the Cacao15kSNP array provides the genotypic data necessary to advance cacao breeding efforts, and advances the goals of achieving an integrated selection platform in the years to come.

## Author contributions

DL, processed samples, analyzed data and wrote the manuscript. CoS, processed samples, analyzed data and wrote the manuscript. GM, processed samples, analyzed data and wrote the manuscript. DR, processed samples, and analyzed data. CaS, processed samples, and analyzed data. FA, processed samples, and analyzed data. FF, analyzed data and wrote the manuscript. KM, processed samples, analyzed data. OC, processed samples, analyzed data. JM, conceived experiment, analyzed data and wrote the manuscript.

### Conflict of interest statement

The authors declare that the research was conducted in the absence of any commercial or financial relationships that could be construed as a potential conflict of interest.

## Supplementary material

Supplementary File 1 R-script for inferring the paternal genotype from Stalin B.

Supplementary Figure 1 Plots of the Matina 1-6 genome assembly position against the linkage map position of the two Stalin populations. The SNP marker position (bp) from the Matina 1-6 genome assembly (y-axis) is plotted against the genetic linkage map position (cM, x-axis) from the Stalin A **(left)** and Stalin B **(right)** linkage maps.

Supplementary Figure 2 Correlation plots of the phenotypic traits for Stalin A. Stalin A correlations between the yield –related and disease resistance traits. The upper diagonal entries show the Pearson correlation coefficient (ρ*)*. The lower diagonal entries show pairwise plots for each trait combination and their corresponding fitted line (in red) of the linear regression. Significance (*H*_0_: ρ = 0) is denoted by ^***^, ^**^, ^*^, “.” for *p* < 0.0001, 0.001, 0.05, 0.10, respectively.

Supplementary Figure 3 Correlation plots of the phenotypic traits for Stalin B. Stalin B correlations between the yield –related and disease resistance traits. The upper diagonal entries show the Pearson correlation coefficient (ρ*)*. The lower diagonal entries show pairwise plots for each trait combination and their corresponding fitted line (in red) of the linear regression. Significance (*H*_0_: ρ = 0) is denoted by ^***^, ^**^, ^*^, “.” for *p* < 0.0001, 0.001, 0.05, 0.10, respectively.

Supplementary Table 1 List of SNPs included on the 15kcacao array and their sequences and genomic positions.

Supplementary Table 2 Linkage map table for the Stalin A population.

Supplementary Table 3 Linkage map table for the Stalin B population.
