# Corrigendum: A Larger Chocolate Chip—Development of a 15K *Theobroma cacao* L. SNP Array to Create High-Density Linkage Maps

**DOI:** 10.3389/fpls.2018.00948

**Published:** 2018-07-16

**Authors:** Donald Livingstone III, Conrad Stack, Guiliana M. Mustiga, Dayana C. Rodezno, Carmen Suarez, Freddy Amores, Frank A. Feltus, Keithanne Mockaitis, Omar E. Cornejo, Juan C. Motamayor

**Affiliations:** ^1^Mars, Inc., Miami, FL, United States; ^2^Estación Experimental Tropical Pichilingue, National Institute of Agricultural Research, Quevedo, Ecuador; ^3^Agricultural Faculty, Technical University of Quevedo, Quevedo, Ecuador; ^4^Universidad Técnica Estatal Quevedo (UTEQ), Quevedo, Ecuador; ^5^Department of Genetics and Biochemistry, Clemson University, Clemson, SC, United States; ^6^Department of Biology and Pervasive Technology Institute, Indiana University Bloomington, Bloomington, IN, United States; ^7^School of Biological Sciences, Washington State University, Pullman, WA, United States

**Keywords:** cacao, SNP, genotyping, QTL, linkage map

In the original article, there was a mistake in Supplemental Table 2 as published online. Supplemental Table 2 was a duplication of Supplemental Table 3. The corrected Supplemental Table 2 is published with this correction article. The authors apologize for this error and state that this does not change the scientific conclusions of the article in any way.

The original article has been updated.

## Conflict of interest statement

The authors declare that the research was conducted in the absence of any commercial or financial relationships that could be construed as a potential conflict of interest.

