# Supplementary material for: A Larger Chocolate Chip—Development of a 15K Theobroma cacao L. SNP Array to Create High-Density Linkage Maps
Source: Front Plant Sci. 2017 Dec 5;8:2008. doi: 10.3389/fpls.2017.02008 (PMC5723429; doi:10.3389/fpls.2017.02008)
Supplement: Supplementary File 1 — R-script for inferring the paternal genotype from Stalin B. [file SupplementaryFile1.PDF]

```
library("reshape2")

#setwd("C:/Users/mustigui/Documents/MARS/STALIN_B")
#setwd("~/sa/Ecuador/data_processing/stuff")

#mom_geno <- read.table("SCA12_15K.txt", sep="", stringsAsFactors=F)
# genos <- read.table("StalinBonly.ped", sep= "", stringsAsFactors=F)
# map <- read.table("StalinBonly.map", sep = "", stringsAsFactors=F)
# genos <- read.table("step1.ped", sep= "", stringsAsFactors=F)
# map <- read.table("step1.map", sep = "", stringsAsFactors=F)
genos <- read.table("step1.Stalin_B_mod.ped", sep= "", stringsAsFactors=F)
map <- read.table("step1.Stalin_B_mod.map", sep = "", stringsAsFactors=F)

tmp = read.table("step1.Stalin_B.txt")
stopifnot(all(genos[,2] %in% tmp[,2]))

# Format progenies
gcols <- seq(7, ncol(genos), by=2)
tgenos <- matrix(NA, nrow=nrow(genos), ncol=length(gcols))
count=1
for (i in gcols)
{
	tmpalleles = cbind(substr(as.character(genos[,i]),1,1),
	                   substr(as.character(genos[,i+1]),1,1))
	tmpalleles <- t(apply(tmpalleles,1,sort))
	tgenos[,count] <- sprintf("%s%s",tmpalleles[,1],tmpalleles[,2])
	count=count+1
}
```

```
#scan("step1.Stalin_B.txt",what=character())

rownames(tgenos) <- genos[,2]

mom.ind = which(genos[,2]=="WG0231160-DNAA08") # mom (TODO: also try out WG0231145-DNAE10 as the mother)
#mom.ind = which(genos[,2]=="WG0231145-DNAE10") # mom (TODO: also try out WG0231145-DNAE10 as the mother)
mom_geno <- tgenos[mom.ind,,drop=FALSE]
tgenos <- tgenos[-mom.ind,]

#all(apply(tgenos,1:2,nchar)==2)
#write.csv(tgenos, "tgenos.csv", row.names=F)
#write.csv(mom, "mom.csv", row.names=F)

################################################################################

models = matrix(c(
        0.25,0.50,0.25, # both heterozygotes
        0.50,0.50,1e-3, # homo/het
        1e-3,0.50,0.50, # het/homo
        1e-3,1.00,1e-3, # opposite homozygotes
        1.00,1e-3,1e-3,  # both homozygous, accounting for sequencing error
        1e-3,1e-3,1.00   # both homozygous, accounting for sequencing error
        ),ncol=3,byrow=TRUE)
```

```
homos.col = c(1,3)
het.col = 2

ngenos = dim(tgenos)[1]
#ff = file("guess_father.log","w")

screwy.inds = c()
missing.char = "0"
dad = matrix(NA, nrow=nrow(mom_geno), ncol=ncol(mom_geno))
best.model = 0
best.models = rep(NA,length(gcols))
seg.dad = seg.mom = rep(FALSE, length(gcols))

for (i in 1:length(gcols))
{
	print(i)
	ualleles = unique(unlist(strsplit(tgenos[,i],"")))
	ualleles = sort(setdiff(ualleles, missing.char))

	mom.alleles = strsplit(mom_geno[,i],"")[[1]]
	mom.allele.count = length(unique(mom.alleles))

	if(any(mom.alleles == missing.char))
	{
		screwy.inds = c(screwy.inds, i)
		next
	}

    if(length(ualleles) == 1){
```

```
			# if there is only one allele in the progeny
			# then, genotype of unknown parent == genotype of known parent
			# NOT accounting for error
			dad[,i] = mom_geno[,i]

		} else {

			theo = sort(unique(apply(expand.grid(ualleles,ualleles),1,function(x)
paste(sort(x),collapse="") )))
			tab = table( factor(tgenos[,i],levels=theo) )
			freq = tab/sum(tab)

			pval =  numeric(nrow(models))
			chisq = numeric(nrow(models))
			for(ii in seq(nrow(models)) ){
				#tmp = chisq.test( as.numeric(freq), p=models[ii,],rescale.p=TRUE)
				#tmp = chisq.test( as.numeric(tab), p=models[ii,],rescale.p=TRUE,
simulate.p.value=TRUE)
				tmp = chisq.test( as.numeric(tab), p=models[ii,],rescale.p=TRUE,
simulate.p.value=F)
				chisq[ii] <- tmp$statistic
				pval[ii] <- tmp$p.value
			}
			best.model = which.min(chisq)
			#sprintf("%0.3f",chisq)

			if( best.model == 1 ){
				# both heterozygotes
				#stopifnot( mom.allele.count==2 )
				if( mom.allele.count==2 ){
```

```
dad[,i] = mom_geno[,i]
#seg.mom[i] <- seg.dad[i] <- TRUE
} else {
screwy.inds = c(screwy.inds, i)
next
}
} else if (best.model == 2 || best.model == 3){
# het/homo or homo/het ->
if( mom.allele.count==2 ){
# dad is homozygous
dad[,i] = names(which.max(freq[homos.col]))
seg.mom[i] <- TRUE
} else {
# dad is heterozygous
dad[,i] = names(which.max(freq[het.col]))
seg.dad[i] = TRUE
}
} else if (best.model == 4) {
# opposite homozygotes
dad.allele = setdiff(ualleles,unique(mom.alleles))
if(length(dad.allele)!=1){
dad[,i] <- NA
screwy.inds = c(screwy.inds, i)
next
} else {
dad[,i] = sprintf("%s%s",dad.allele,dad.allele)
}

} else {
```

```
                # both same homozygous, accounting for sequencing error
                dad[,i] = mom_geno[,i]
            }
        }

        stopifnot( !is.na(dad[,i]), !is.na(mom_geno[,i]) )
        best.models[i] <- best.model

        # #print(table(tgenos[,i]))
        # cat(names(table(tgenos[,i])),"\n",file=ff)
        # cat(table(tgenos[,i]),"\n",file=ff)
        # cat(sprintf("Mom\tDad\n%s\t%s\n",mom_geno[1,i],dad[1,i]), file=ff)
        # cat(sprintf("Best model: %d\n",best.model), file=ff)

}
#flush(ff); close(ff)


# Check for strange patterns of segregation in genotypes
# of both the (inferred) father and mother:

# Counts:
sdad = (which(seg.dad)); length(sdad) # 1494
smom = (which(seg.mom)); length(smom) # 1041
both.inds = which(seg.dad | seg.mom) # 2535

#
snpname = map[,2]
snptokens = sapply(strsplit(snpname,"s"),function(x) as.integer(gsub("[^0-9]","",x)))
```

```
lgs = snptokens[1,]
pos = snptokens[2,]

# father
seg.inds = which(seg.dad)
mom.inds = which(seg.mom)
dad.rng = numeric(10)
mom.rng = numeric(10)
for(ii in 1:10){
	brkpts = seq(0,45000000,by=5000000)

	par(mfrow=c(2,1))
	sub.inds = seg.inds[which(lgs[seg.inds]==ii)]
	#xd = hist(pos[sub.inds],xlim=c(0,(max(pos[which(lgs==ii)]) + 100000)), breaks=100, freq=FALSE)
	xd = hist(pos[sub.inds], breaks=brkpts, freq=FALSE)
	dad.rng[ii] <- diff(range(pos[sub.inds]))

	sub.inds = mom.inds[which(lgs[mom.inds]==ii)]
	#xm = hist(pos[sub.inds],xlim=c(0,(max(pos[which(lgs==ii)]) + 100000)), breaks=100, freq=FALSE)
	xm = hist(pos[sub.inds], breaks=brkpts, freq=FALSE)
	mom.rng[ii] <- diff(range(pos[sub.inds]))

	dad.inds = seg.inds[which(lgs[seg.inds]==ii)]
	both.tmp = both.inds[which(lgs[both.inds] == ii )]
	#sub.inds = both.tmp[which(seg.dad[both.tmp])]
	#krtest = kruskal.test(pos[both.tmp],seg.dad[both.tmp])
	#krtest = ks.test( pos[sub.inds],pos[dad.inds] )
	krtest = fisher.test(xm$counts,xd$counts,simulate.p.value=TRUE,B=1000000)
```

```
        cat(sprintf("LG %02d - %0.8f\n",ii,krtest$p.value))
}


# plot(dad.rng,mom.rng)
# identify(dad.rng,mom.rng,1:10)




# Final QC
all(which(is.na(dad[1,])) == screwy.inds)  # TRUE?


rownames(dad) <-"unknown"
fixed = rbind(
                mom_geno,
                dad,
                tgenos
            )
colnames(fixed) <- map[,2]
fixed = fixed[,-screwy.inds]



# NB -> (2017-03-27) I had to change the missing value code in step1.Stalin_B_mod2.ped.  It came out as NA, but should be 0.  The practical implications of this should have been minimal, because plink's default behavior is to convert the NA values (i.e., the rarest of the 3 alleles at what plink sees as a "triallelic" locus) to missing values.
```
