# Supplementary figures and images for: A Larger Chocolate Chip—Development of a 15K Theobroma cacao L. SNP Array to Create High-Density Linkage Maps
Source: Front Plant Sci. 2017 Dec 5;8:2008. doi: 10.3389/fpls.2017.02008 (PMC5723429; doi:10.3389/fpls.2017.02008)

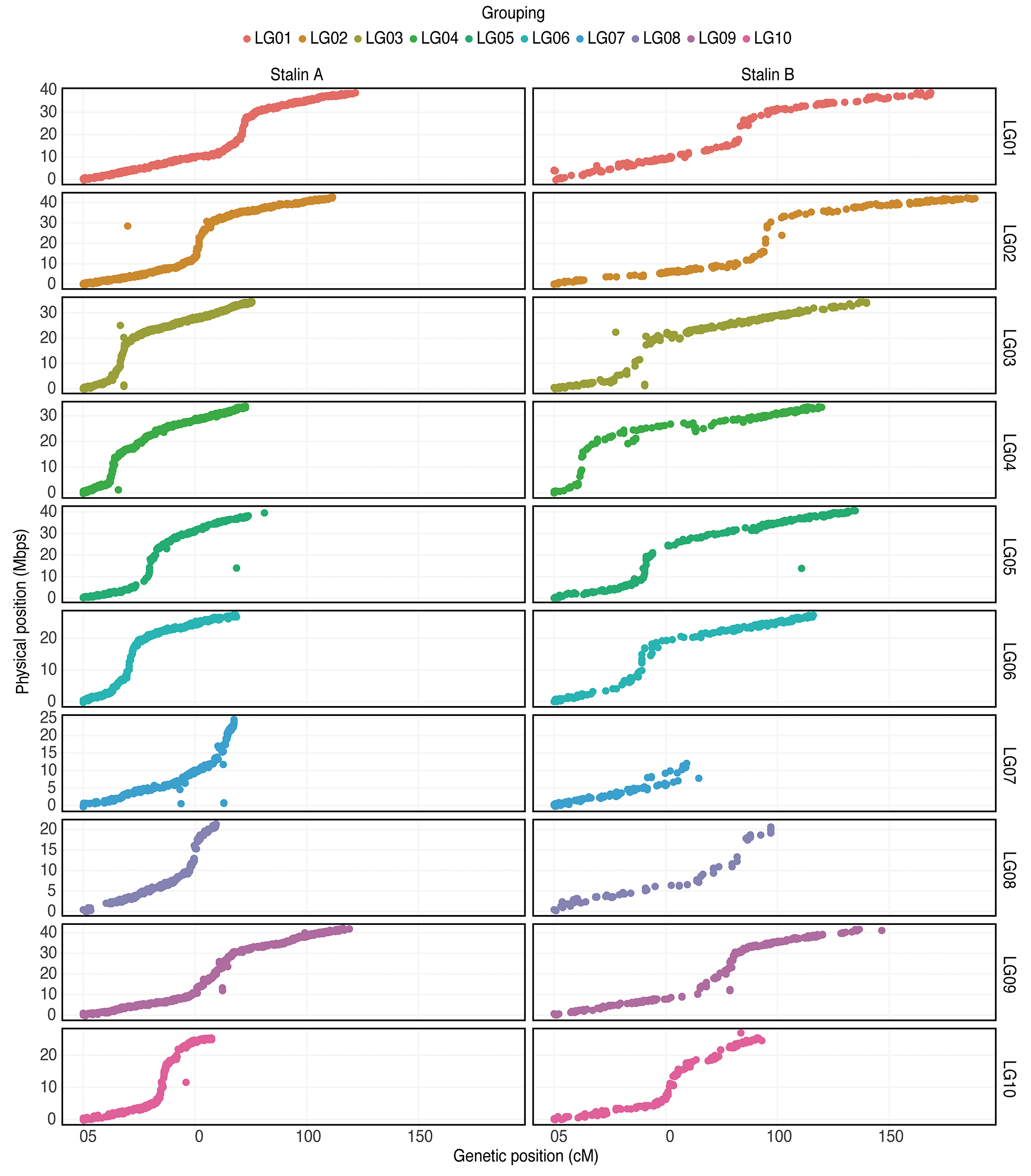

Supplement: Supplementary Figure 1 — Plots of the Matina 1-6 genome assembly position against the linkage map position of the two Stalin populations. The SNP marker position (bp) from the Matina 1-6 genome assembly (y-axis) is plotted against the genetic linkage map position (cM, x-axis) from the Stalin A (left) and Stalin B (right) linkage maps. [file SupplementaryFigure1.TIF]

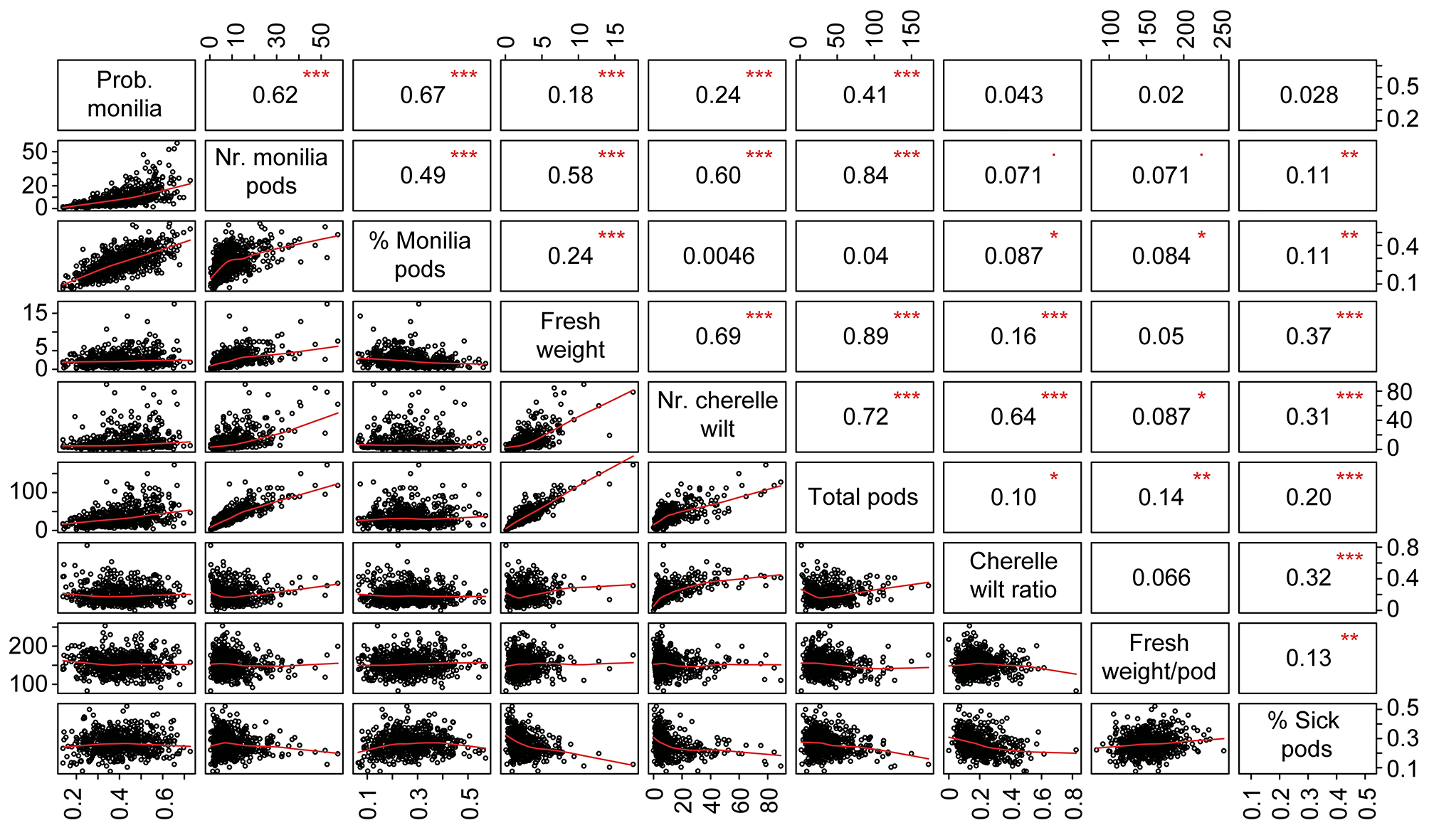

Supplement: Supplementary Figure 2 — Correlation plots of the phenotypic traits for Stalin A. Stalin A correlations between the yield –related and disease resistance traits. The upper diagonal entries show the Pearson correlation coefficient (ρ). The lower diagonal entries show pairwise plots for each trait combination and their corresponding fitted line (in red) of the linear regression. Significance (H0: ρ = 0) is denoted by ***, **, *, “.” for p < 0.0001, 0.001, 0.05, 0.10, respectively. [file SupplementaryFigure2.TIF]

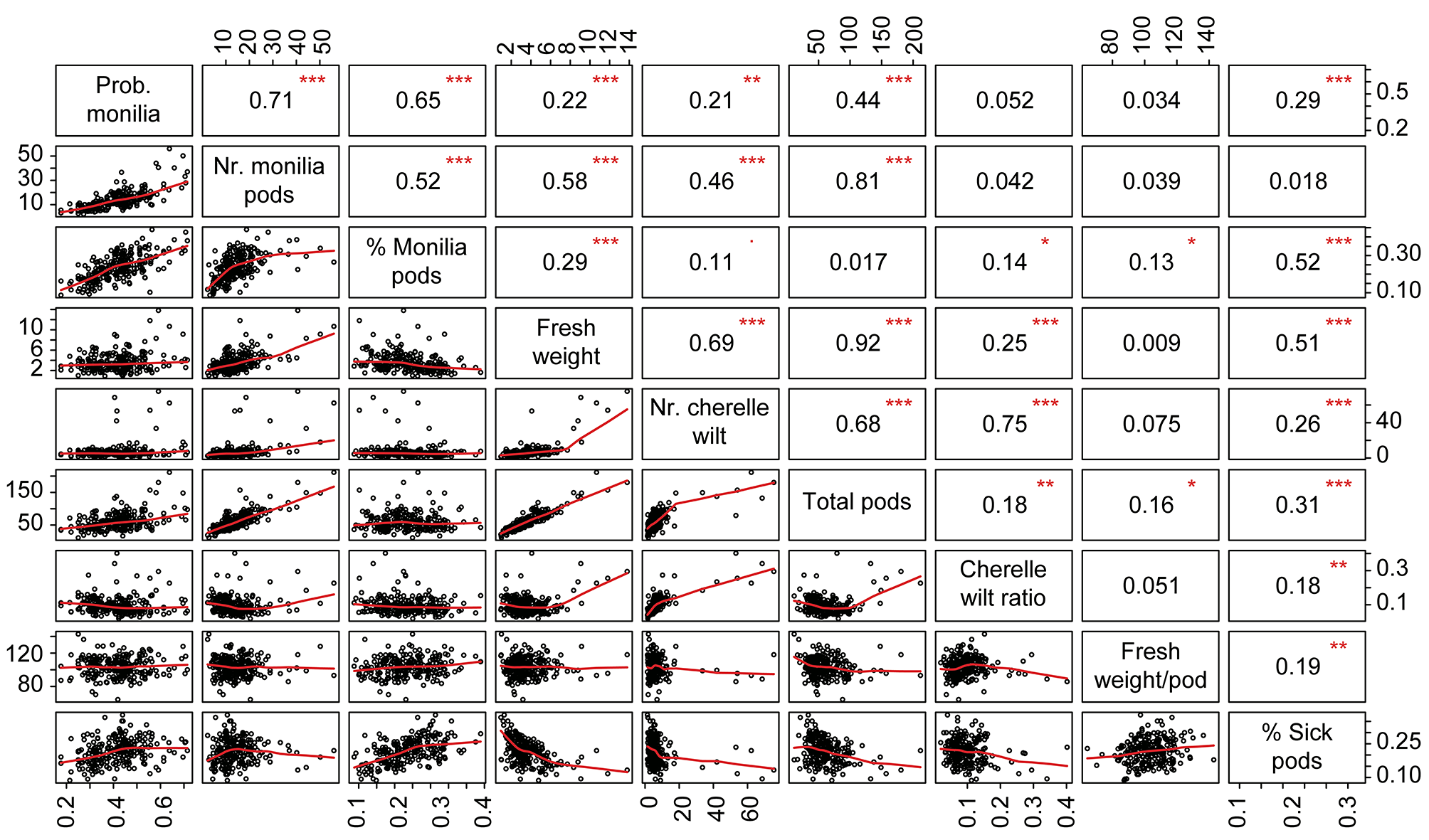

Supplement: Supplementary Figure 3 — Correlation plots of the phenotypic traits for Stalin B. Stalin B correlations between the yield –related and disease resistance traits. The upper diagonal entries show the Pearson correlation coefficient (ρ). The lower diagonal entries show pairwise plots for each trait combination and their corresponding fitted line (in red) of the linear regression. Significance (H0: ρ = 0) is denoted by ***, **, *, “.” for p < 0.0001, 0.001, 0.05, 0.10, respectively. [file SupplementaryFigure3.TIF]
